# Potential Distribution and Key Factors of *Dasyhippus barbipes* (Orthoptera: Acrididae: Gomphocerinae) in China Under Climate Change Scenarios

**DOI:** 10.3390/insects17060616

**Published:** 2026-06-11

**Authors:** Qian Wang, Fuyuan Ta, Fangzheng Yue, Heting Ma, Ding Yang

**Affiliations:** 1 College of Forestry and Grassland, Qinghai University, Xining 810000, China; wangqian@qhu.edu.cn (Q.W.);; 2 Center for Biological Disaster Prevention and Control, National Forestry and Grassland Administration, Shenyang 110034, China17741334015@163.com (H.M.)

**Keywords:** grasshopper, *Dasyhippus barbipes*, MaxEnt, climate change, habitat suitability, distribution prediction

## Abstract

*Dasyhippus barbipes* (Fischer von Waldheim, 1846) is an early dominant grasshopper in Chinese grasslands, and understanding its climatic niche is important for monitoring and early warning. Based on nationwide field surveys conducted from 2019 to 2024, 732 filtered occurrence records were used with an optimized MaxEnt model parameters (rm = 1.5, fc = LQ) to predict the current and future suitable habitat of *D. barbipes* in China. The model performed well (mean AUC = 0.962, mean TSS = 0.924). The mean temperature of the coldest quarter and precipitation seasonality were the most important limiting variables, while elevation and human footprint also contributed to habitat suitability. Under current climate conditions, suitable habitat is concentrated mainly in central and eastern Inner Mongolia, with additional suitable areas in the eastern Tianshan Mountains of Xinjiang and the central Qilian Mountains of Gansu; the highly suitable habitat covers 6.57 × 10^4^ km^2^. Under all future climate scenarios, suitable habitat is projected to shift northward, and stable areas are mainly located in eastern Inner Mongolia. Habitat changes become more pronounced by the 2090s. These results improve understanding of the spatiotemporal dynamics of *D. barbipes* under climate change and provide a basis for future monitoring and management.

## Simple Summary

*Dasyhippus barbipes* (Fischer von Waldheim, 1846) is a common grasshopper in Chinese grasslands and can contribute to grassland pest damage. Understanding where this species can live now and how its range may change as the climate warms is important for surveillance and early warning. In this study, we used 732 occurrence records collected from field surveys and optimized MaxEnt model parameters to estimate current and future suitable habitats across China. The model showed that winter temperature, variation in precipitation, elevation, and human activity were the main factors affecting distribution. Suitable areas are currently concentrated in central and eastern Inner Mongolia, with smaller suitable areas in parts of Xinjiang and Gansu. Under future climate scenarios, the suitable range is expected to shift northward and to contract in some regions, especially by the 2090s. These results can help target future monitoring and management of grassland grasshoppers.

## 1. Introduction

The Sixth Assessment Report of the Intergovernmental Panel on Climate Change (IPCC) indicates that climate warming has significantly affected regional and global ecosystems [1]. As global warming continues to intensify, the stability and security of ecosystems face further threats, with particularly profound impacts on ecosystem functions, species diversity, and service capacity in arid and semi-arid regions [2,3]. Grasslands, as a critical component of terrestrial ecosystems, account for approximately 40% of the global land area [4,5]. They not only serve as the foundation for livestock production but also act as important carriers of grassland culture, while playing key roles as ecological barriers. Grasslands provide essential ecosystem services and functions, including climate regulation, water and soil conservation, water retention, carbon sequestration and sink enhancement, as well as the maintenance of biodiversity and gene pools [6,7]. The total area of natural grasslands in China reaches 400 million hectares, ranking second in the world and primarily distributed across arid and semi-arid regions [8]. Among these, over 90% are experiencing varying degrees of degradation [9], with severely degraded grasslands accounting for more than 60% of the total [10]. Under the combined pressures of climate change and human activities, the occurrence patterns and geographical distributions of pests and diseases have undergone profound changes, posing substantial threats to the ecological security of grasslands [11,12].

Grasshoppers are the most important phytophagous invertebrates and primary consumers in most grassland ecosystems, occupying a significant ecological niche. Their occurrence and distribution are closely related to environmental factors and vegetation conditions [13]. Studies have shown that droughts and warm years are prone to grasshopper outbreaks [14,15]. Grasshopper plagues, together with floods and droughts, are recognized as the three major natural disasters in China. Characterized by their outbreak potential, destructiveness, and migratory capacity, grasshopper infestations cause severe damage to livestock production and the grassland ecological environment, posing serious threats to the livelihoods of herders and farmers [16].

*Dasyhippus barbipes* (Fischer von Waldheim, 1846), an early-season dominant grasshopper species in China’s grassland regions [17], belongs to Orthoptera: Acrididae: Gomphocerinae: Gomphocerini. It is small in body size, has weak migratory and dispersal abilities, produces one generation per year, and overwinters as eggs. In China, it is mainly distributed in grassland areas of Jilin, Inner Mongolia, Gansu, and other provinces [18]. This grasshopper feeds on gramineous forage grasses, preferring plants such as *Leymus chinensis* and *Leymus secalinus* [18]. It plays a supplementary role in grasshopper plagues and often acts together with other grasshopper species to intensify ecological pressure on grasslands. In recent years, *D. barbipes* has consistently maintained a high incidence, with large population densities and strong reproductive capacity observed in lightly degraded or grazed grasslands [18,19]. As an important pest of forage grasses in China’s grassland ecosystems, it has received widespread attention [19].

Species distribution models originated with the development and application of the Bioclim model, which integrates species distribution data and associated environmental factors to characterize species’ ecological niche requirements and predict potential habitat suitability [20]. Building on this foundation, species distribution models use actual species occurrence data along with relevant environmental variables, combined with algorithms to identify environmental factors associated with species occurrence under climate change scenarios, thereby enabling the projection of potential distribution trends under specific spatiotemporal conditions in the future [21,22]. Among these, the Maximum Entropy model (MaxEnt) has been widely adopted in species distribution prediction research due to its advantages in efficient sample utilization and high predictive accuracy [23,24]. By elucidating the relationships between grasshoppers and environmental factors, models linking grasshopper distribution to habitat suitability can be constructed to delineate suitable areas for grasshopper occurrence and to support forecasting of outbreak trends.

Currently, the overall distribution pattern of *D. barbipes* in China under climate change and human activities, along with its key limiting factors, remains unclear. To address this knowledge gap, this study employs an optimized MaxEnt model to identify key limiting factors of suitable habitats for *D. barbipes* and to predict their range dynamics under future climate scenarios. The findings provide a scientific basis for grassland authorities to optimize spatial allocation of priority control areas and deploy plant protection resources efficiently, thereby enhancing the precision of pest management and delivering data support for monitoring and accurate forecasting of grassland grasshoppers in China.

## 2. Materials and Methods

### 2.1. Sources and Screening of D. barbipes Distribution Data

**Figure 1:**
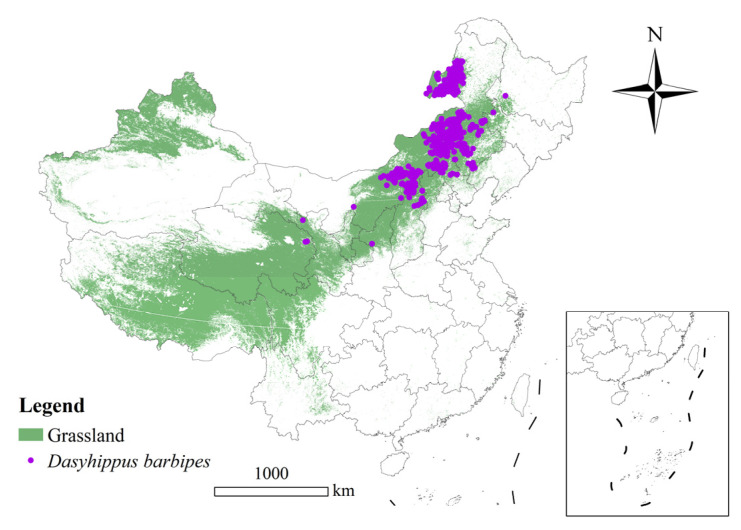
Distribution of *D. barbipes* species occurrence points.

In accordance with the agricultural industry standard of the People’s Republic of China (NY/T 1578-2007, Specification for Grasshopper Survey in Grasslands) [25], a nationwide survey was conducted in grassland regions from 2019 to 2024 to document the species and distribution of grassland grasshoppers. A total of 4280 occurrence points of *D. barbipes* were recorded. The survey data were obtained from the Biological Disaster Prevention and Control Center of the National Forestry and Grassland Administration. To minimize sampling bias and reduce the risk of model overfitting, and referring to similar thresholds used in studies of grasshoppers with comparable dispersal abilities, we thinned the occurrence points using ENMTools v1.3 to ensure that no two occurrence points were located within a 10 km radius [18,26]. Ultimately, 732 valid distribution points were retained, as shown in Figure 1 and Table S1. All species were initially identified by staff with an academic background in entomology primarily based on the Fauna Sinica: Insecta, and the identification results were subsequently verified by relevant taxonomic experts.

### 2.2. Environmental Variables

#### 2.2.1. Sources of Climate Data

Climate data were obtained from the WorldClim database (WorldClim version 2.1, http://www.worldclim.org/, accessed on 9 January 2024), comprising 19 bioclimatic variables at a spatial resolution of 30 arcseconds (approx. 1 km at the equator). For future climate scenarios, the BCC-CSM2-MR climate model under the Coupled Model Intercomparison Project Phase 6 (CMIP6) was selected [27]. Data for the 2050s, 2070s, and 2090s were used under three Shared Socioeconomic Pathways (SSPs): SSP126 (low greenhouse gas emissions, with CO_2_ emissions reaching net zero around 2075), SSP245 (intermediate greenhouse gas emissions, with CO_2_ emissions remaining at current levels until 2050 and then declining but not reaching net zero by 2100), and SSP585 (very high greenhouse gas emissions, with CO_2_ emissions tripling by 2075) [1,27]. Compared to the previous generation (Coupled Model Intercomparison Project Phase 5, CMIP5), the BCC-CSM2-MR model shows marked improvement in simulating the spatial distribution of mean annual precipitation over China [27].

#### 2.2.2. Other Data

Topography influences local microclimate (e.g., temperature, precipitation, solar radiation) and soil moisture, thereby indirectly determining vegetation types and grasshopper habitat suitability. Elevation serves as an effective proxy for temperature gradients, while slope and aspect affect the redistribution of heat and water, which in turn influence grasshopper oviposition and nymphal survival conditions. In this study, topographic data were obtained from the Digital Elevation Model (DEM) provided by the Geospatial Data Cloud (https://www.gscloud.cn/, accessed on 9 January 2024), with a spatial resolution of 30 arcseconds. Elevation, slope, and aspect were extracted using ArcGIS 10.2. These topographic conditions showed little change during the study period; therefore, we assumed that they did not vary significantly over the study period.

Soil physicochemical properties directly affect the overwintering survival rate of grasshopper eggs and vegetation growth [16,22]. In this study, soil data were obtained from the Harmonized World Soil Database (HWSD) published by the Food and Agriculture Organization of the United Nations (FAO), at a resolution of 30 arcseconds. Human activities such as grazing and infrastructure construction can alter grassland vegetation cover and microclimate, thereby affecting grasshopper population dynamics. The Human Footprint (HFP) data, which is a composite indicator reflecting the overall intensity of anthropogenic disturbance, were obtained from the FigShare repository (https://figshare.com/) at the same resolution of 30 arcseconds (Table 1).

Different land use types provide different food resources and habitat conditions. Grasshoppers show a preference for grassland [14]. Incorporating the global Land Use and Land Cover (LULC) projection dataset helps improve the model’s ability to capture habitat heterogeneity across different time periods and scenarios. This dataset provides land use projections with a spatial resolution of 30 arcseconds for the period 2020–2100 under the SSP-RCP scenarios and includes six land use types: forest, grassland, cropland, water, built-up land, and barren land [28].

### 2.3. Environmental Variable Screening

**Figure 2:**
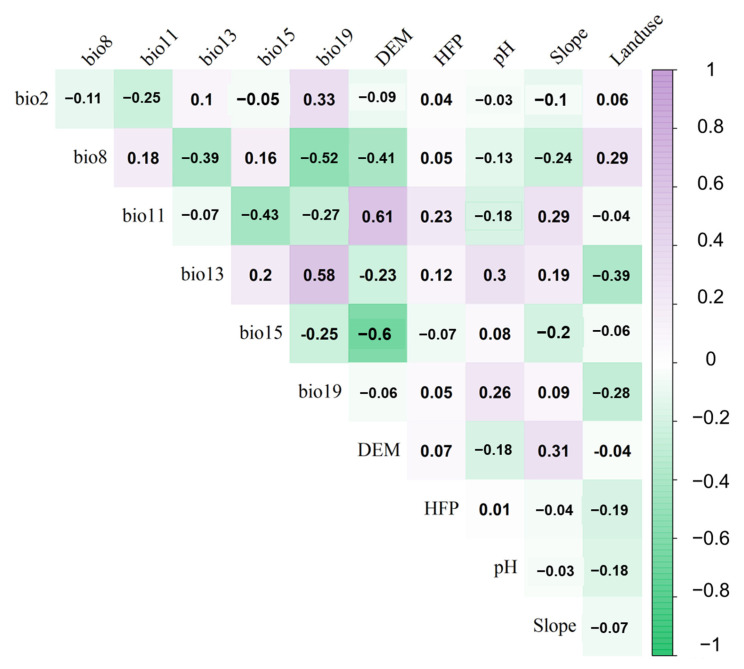
Spearman correlation among environmental variables.

Due to spatial correlations among environmental variables, model overfitting may occur, ultimately affecting the prediction accuracy. To improve reliability, environmental variables were screened accordingly to remove spatial correlations. First, all variables together with the filtered occurrence points were imported into MaxEnt v3.4.4 for preliminary analysis to obtain the contribution percentage of each variable [29]. Subsequently, 25 environmental variables were extracted at the grasshopper occurrence locations using the “Extract Multi Values to Points” tool in ArcGIS 10.2 (Table S2). Spearman’s correlation test was then performed in R 4.1.2 using the “cor.test” function from the base “stats” package version 4.1.2 to assess correlations among all variables, and a correlation plot was generated. When |*r*| ≥ 0.85, the variable with the higher contribution was retained for modeling (Figure 2 and Table 1).

### 2.4. MaxEnt Model Optimization and Execution

To further improve model accuracy, the ENMeval package (version 2.0.3 in R version 4.1.2) was used to tune the hyperparameters of the model [30]. The ENMeval package splits the dataset using the default “checkerboard2” spatial partitioning method [30]. By adjusting the regularization multiplier (RM) and feature combination (FC) parameters in the MaxEnt model, the complexity of the model under various parameter settings was analyzed, and the parameter combination yielding the lowest complexity was selected [31]. The parameter combination with the lowest AICc value was selected as the optimal model [6]. In this study, RM was set from 0.5 to 4 in increments of 0.5, and six feature combinations (FC) were applied: L, LQ, H, LQH, LQHP, and LQHPT [30,31].

The number of background points was set to 10,000, the random seed was set to 1, and the “Do clamping” option was enabled (extrapolation was not allowed). The output format was set to Logistic and saved as .asc files, with all other parameters left as default [32]. The final prediction map was produced by averaging the 10 replicate logistic rasters. The predictive performance was evaluated using the receiver operating characteristic (ROC) curve, with the area under the ROC curve (AUC) serving as an indicator of prediction accuracy. AUC values range from 0 to 1, with higher values indicating greater model precision [33]. The true skill statistic (TSS) ranges from −1 to 1, where higher TSS values indicate better agreement between observed and predicted values, reflecting stronger model performance [34].

### 2.5. Suitable Habitat Classification and Spatiotemporal Dynamics

The modeling results were imported into ArcGIS 10.2 software, and the maximum training sensitivity plus specificity (MTSS) was used to determine the suitable habitat threshold. Subsequently, the natural breaks method was used to classify suitable habitats into four categories: non-suitable habitat (0–0.15), low-suitability habitat (0.15–0.3), medium-suitability habitat (0.3–0.6), and high-suitability habitat (0.6–1.0). The area of each suitability class was calculated using the “Calculate Geometry” tool in ArcGIS based on projected coordinates (Albers equal-area projection). Subsequently, the prediction results were binarized to distinguish suitable from non-suitable habitats [35]. The SDMtoolsbox version 2.5 was then used to extract stable, expansion, and contraction zones [36], and the corresponding areas were calculated relative to the current climate scenario.

## 3. Results

### 3.1. Model Optimization and Evaluation, and Current Suitable Habitat

**Figure 3:**
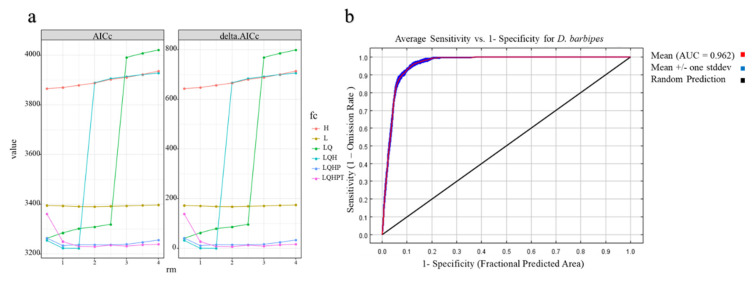
Model Optimization and Evaluation. (**a**) Model tuning results; (**b**) average receiver operating characteristic (ROC) curves from 10 bootstrap replicates.

According to the calculation results from the “ENMeval” package, the AIC value was lowest when rm = 1.5 and fc = LQ (Figure 3a, Table S3). These parameters were therefore set as the optimal parameters for the MaxEnt model in this study. After 10 repeated runs, the average AUC value was 0.962 and the average TSS value was 0.924, indicating that the model achieved excellent accuracy and stability and can reliably predict the distribution of *D. barbipes* in China (Figure 3b, Table S4).

**Figure 4:**
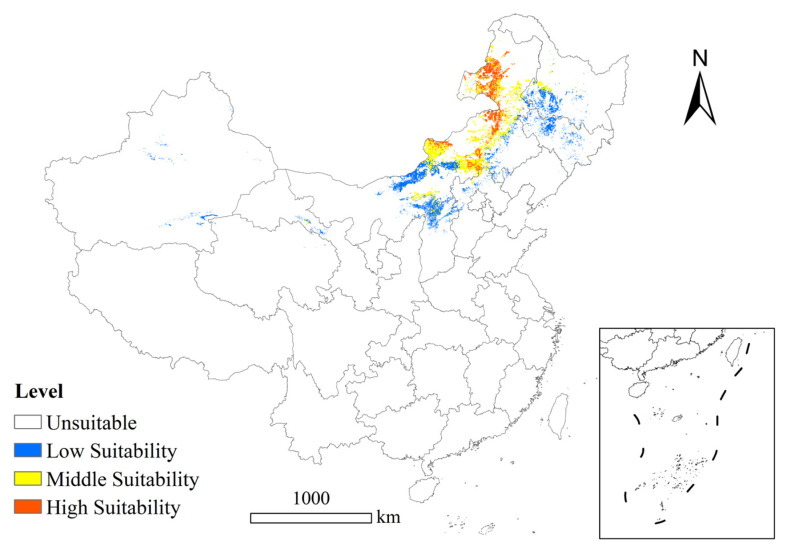
Current suitable habitat distribution and proportional area of suitable habitats.

Based on the suitability classification, the habitat of *D. barbipes* was reclassified into five suitability levels (Figure 4). The suitable habitats of *D. barbipes* are mainly distributed in the high-latitude regions of China, with some areas also found in the mountainous regions of the northwest. The total suitable habitat area is 3.13 × 10^5^ km^2^, of which the highly suitable habitat covers 6.57 × 10^4^ km^2^, the moderately suitable habitat covers 1.20 × 10^5^ km^2^, and the low-suitability habitat accounts for 40.64% of the total area.

### 3.2. Key Limiting Factors

Taking into account the importance of environmental factors, this study analyzed the response relationships between the distribution probability of *D. barbipes* and environmental factors, the contribution rates of environmental variables in MaxEnt, the suitable environmental conditions, and the key factors influencing the species. Among the variables used for modeling, bio11 (mean temperature of the coldest quarter) was the most significant factor influencing the spatial distribution of *D. barbipes*, with a contribution rate of 83.5%. Bio15 (precipitation seasonality), DEM (elevation), and HFP (Human Footprint Index) also played important roles, with a combined contribution of 14.4% (Table 2).

**Figure 5:**
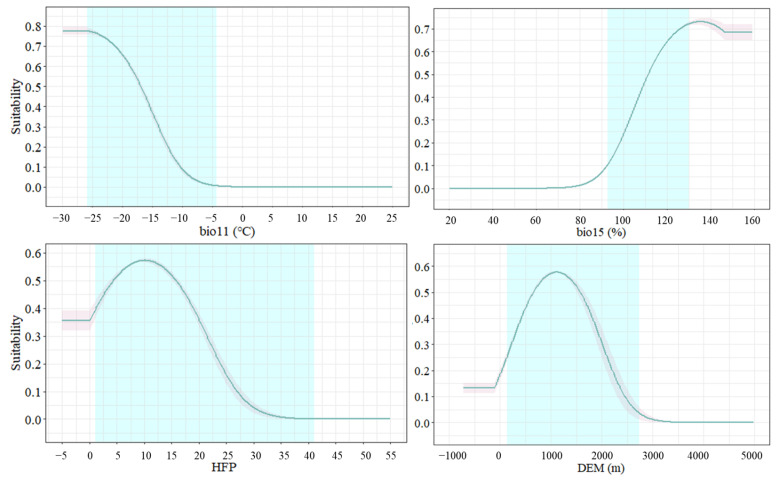
The relationship between the distribution probability of *D. barbipes* and only a single major environmental factor. The purple shaded area around the curve represents the mean ± one standard deviation of the 10 prediction results, reflecting the uncertainty range of the model predictions. The light blue shaded area indicates the actual range of occurrence points along the *x*-axis.

Based on the response curves between each variable and the distribution probability of *D. barbipes*, the probability of occurrence showed a monotonic decreasing relationship with bio11 within the range of −25 °C to 0 °C, reaching 0.78 when the mean temperature of the coldest quarter was below −25 °C (Figure 5). The relationship between bio15 and occurrence probability followed a parabolic pattern within the range of 58% to 145%, with the highest probability occurring at 135.13%, indicating that greater intra-annual precipitation variability is associated with a higher probability of *D. barbipes* occurrence. DEM showed a parabolic relationship with occurrence probability at elevations below 3000 m, with the highest probability observed at 1106.34 m, suggesting that an elevation of around 1100 m is the most suitable for *D. barbipes*. The occurrence probability of *D. barbipes* initially increased and then decreased with increasing HFP, peaking at a Human Footprint Index value of 10 and dropping to zero when the index exceeded 25.

### 3.3. Potential Distribution of D. barbipes Under Future Climate Scenarios

**Figure 6:**
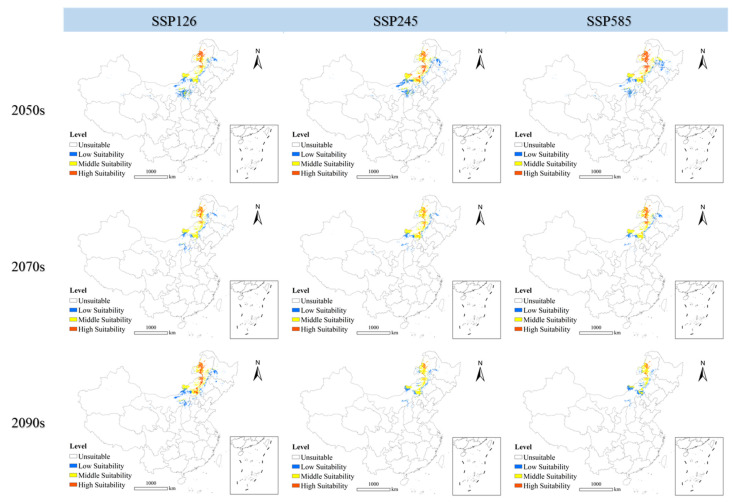
Distribution map of *D. barbipes* under future scenarios.

The MaxEnt model was used to predict the potential suitable areas for *D. barbipes* under the SSP126, SSP245, and SSP585 scenarios for the 2050s, 2070s, and 2090s. The results indicate that under future climate scenarios, *D. barbipes* will be mainly distributed in the high-latitude grassland regions of China, one of the areas most sensitive to climate change (Figure 6, Table 3), and highlight the adverse effects of climate warming on its distribution. Under the SSP126 scenario, the low-suitability habitat area of *D. barbipes* is projected to peak in the 2070s, while under the SSP245 and SSP585 scenarios, it is projected to shrink to its minimum in the 2090s. The medium-suitability habitat area is expected to reach its maximum in the 2070s across all scenarios, then decrease to its minimum in the 2090s. Under the SSP126 and SSP245 scenarios, the high-suitability habitat area is projected to increase to its maximum in the 2090s, whereas under the extreme greenhouse gas emission scenario (SSP585), the high-suitability habitat area is projected to decrease substantially.

### 3.4. Spatiotemporal Dynamics of Suitable Habitats

**Figure 7:**
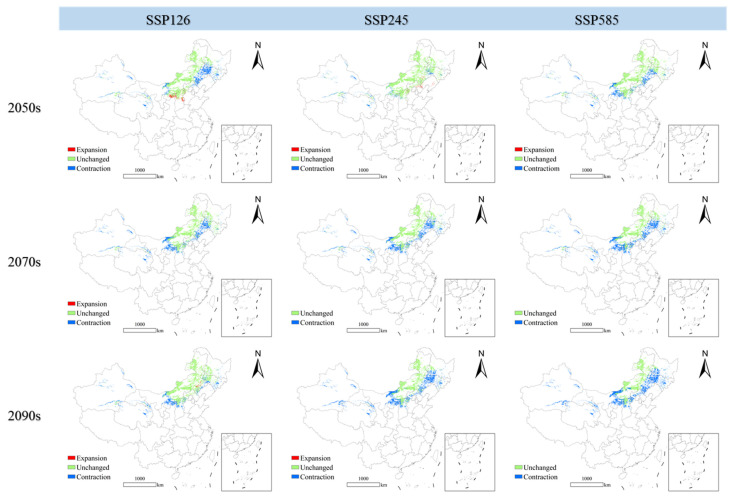
Spatiotemporal dynamics of suitable habitats for *D. barbipes* under different scenarios.

The geographical distribution dynamics of *D. barbipes* under different climate scenarios are shown in Figure 7. The results indicate that future climate warming will negatively affect the suitable habitats of *D. barbipes*. Under all scenarios, the suitable habitats of *D. barbipes* shift northward, with the stable areas mainly located in eastern Inner Mongolia. Compared with the 2050s, more pronounced changes are observed in the 2090s, by which time suitable habitats in the central and western regions have almost disappeared.

Under the SSP126 scenario, the stable areas are relatively extensive, suggesting minimal changes in suitable habitats compared to current conditions, with even some expansion areas appearing. This indicates that even under a low greenhouse gas emission scenario, suitable habitats still show a degree of reduction. However, under the SSP245 scenario, the contraction area increases significantly, with almost no expansion areas observed. Under the SSP585 scenario, the contraction area continues to expand, indicating that under this high-emission scenario, the suitable habitats of *D. barbipes* will be further reduced.

## 4. Discussion

### 4.1. Evaluation of MaxEnt Model Prediction Results

In recent years, the MaxEnt model has become one of the most widely used species distribution models due to its high predictive accuracy [37]. In this study, the model was used to predict the potential suitable distribution of *D*. *barbipes* in China. However, model complexity significantly affects its transferability, and substantial differences in prediction results have been observed between default and optimized parameters [38]. The Akaike Information Criterion (AIC) effectively balances model complexity with goodness of fit [6]. Accordingly, this study found that the AIC value was minimized when a low regularization multiplier (rm = 1.5) and a simplified feature combination (consisting only of linear (L) and quadratic (Q) features) were adopted, and these were therefore determined as the optimal model parameters. Previous studies have shown that when the sample size exceeds 90, the MaxEnt model can achieve high accuracy and stability, with the standard deviation of the AUC value remaining below 0.05, ensuring robust and reliable predictions [39]. In this study, the mean AUC and TSS values of the model exceeded 0.90, indicating “excellent” predictive performance.

### 4.2. Key Environmental Variables Affecting the Distribution of D. barbipes

There are many species of grasshoppers in grasslands, and they are highly sensitive to factors such as climatic conditions, plant nutrient status, grazing activities, and vegetation changes in the grassland [18]. The evaluation of environmental variables in this study indicates that the mean temperature of the coldest quarter (bio11), the coefficient of precipitation variation (bio15), elevation (DEM), and human footprint (HFP) are the main factors influencing the distribution of *D. barbipes*. The distribution probability of *D. barbipes* peaks when the mean temperature of the coldest quarter is below −25 °C, but drops to its lowest when the mean temperature exceeds 0 °C. This may be related to the biological characteristics of *D. barbipes* eggs. Studies have shown that temperature increase only accelerates the overall development of *D. barbipes* eggs by less than one instar [40], suggesting that *D. barbipes* may have adapted to relatively cold regions, requiring cold stimulation to terminate egg diapause and induce synchronized hatching. This is similar to the adaptation of *Cardiodactylus guttulus* to the warm winter environment in subtropical regions [41], but further research is needed for confirmation. Precipitation not only affects the host plants of grasshoppers but also plays an important role in the grasshoppers themselves [42]. The occurrence of grasshopper plagues is highly correlated with precipitation, while showing only a weak correlation with temperature; under dry and relatively cold climatic conditions, grasshopper population densities increase significantly after several years [43]. Since the occurrence of grasshoppers is synchronized with changes in vegetation growth, variations in grassland vegetation growth and habitat vegetation caused by precipitation changes can also alter grasshopper community structure [44]. In this study, the greater the coefficient of precipitation variation—i.e., the more uneven the precipitation distribution within a year—the higher the distribution probability of *D. barbipes*.

Altitude is a major topographic factor affecting the distribution of *Gomphocerus licenti* (Acrididae: Gomphocerinae: Gomphocerini) in the central and eastern parts of the Hexi Corridor in China [45]. In this study, elevation also had a certain effect on the distribution of *D. barbipes*, and it was found that the distribution probability of *D. barbipes* was highest at an elevation of approximately 1100 m. Human activities are also important factors influencing grasshopper distribution. [46]. This study used human footprint data to reflect the impact of human activities on grasshopper distribution, with the highest distribution probability of *D. barbipes* occurring in areas of low human activity intensity (index = 10).

### 4.3. Spatial Dynamics of Suitable Habitat for D. barbipes Under Current and Future Climate Scenarios

Climate warming not only alters temperature and precipitation patterns within ecosystems but also profoundly affects the life history, reproductive rates, and migratory behavior of pests [47]. Under current climatic conditions, the suitable habitat for *D. barbipes* is mainly distributed in the central and eastern grassland regions of Inner Mongolia, with additional suitable areas in the central Qilian Mountains of Gansu. These regions are characterized by severe winters and uneven precipitation, with human disturbance primarily in the form of grazing—a pattern that aligns with the key variables influencing the distribution of *D. barbipes*.

As global warming continues, the distribution ranges of pests and diseases are undergoing unprecedented changes [48]. Overall, the total suitable area for *D. barbipes* is projected to decrease by the 2090s compared to the present. While the distribution ranges of most insect species are expected to expand [49], some species with specific habitat requirements will experience range contractions. Insects respond to climate warming by shifting toward higher altitudes and latitudes, leading to population increases in higher-latitude regions for certain species [50]. The results of this study similarly indicate that by the 2090s, under all scenarios, the distribution of *D. barbipes* will shift toward higher latitudes, while suitable habitats in the eastern Tianshan Mountains of Xinjiang and the central Qilian Mountains of Gansu will disappear. Moreover, as greenhouse gas emissions increase, the changes in *D. barbipes* distribution vary across different time periods, with range contractions occurring mainly in low-latitude regions and the area of range expansion in China decreasing over time. Changes in suitable habitat will have profound impacts on the population dynamics of *D. barbipes* and the associated ecosystem services. Such range contractions may concentrate populations into smaller areas, thereby intensifying local pressures [51]. Therefore, it is necessary to strengthen monitoring and early warning systems in high-latitude regions and establish monitoring stations to ensure timely control of *D. barbipes* population dynamics.

### 4.4. Pest Management Recommendations

The high-suitability areas of *D. barbipes* predicted in this study are mainly concentrated in the central and eastern grasslands of Inner Mongolia. These areas should be designated as priority monitoring zones, where field surveys should be intensified during the egg-hatching period in spring and the nymphal stage, combined with meteorological conditions, to issue short-term outbreak warnings. Furthermore, the mean temperature of the coldest quarter (bio11) was identified as the key factor governing the distribution, suggesting that climate warming may alter the conditions for egg diapause termination, thereby affecting the timing of population outbreaks. Therefore, it is recommended that changes in winter low temperatures be incorporated into a long-term early warning framework, and that a dynamic prediction model based on temperature thresholds be established.

### 4.5. Limitations and Future Directions

However, future soil conditions may differ from the present, and our predictions assume constant non-climatic variables; therefore, the results should be interpreted with caution. This study also has inherent limitations. Factors such as interspecific interactions, natural enemy effects, and dispersal ability may significantly influence the actual distribution of species in natural ecosystems [52]. In addition, to ensure transparency, we have added a statement in the Discussion acknowledging that minor deviations may arise due to potential misidentification of closely related species. Consequently, the ecological niche predicted in this study may be broader than the actual niche. Future efforts should aim to build models incorporating as many relevant influencing factors as possible and adopt multi-model ensemble averaging to reduce uncertainty, thereby providing more reliable decision support for the precise management of grassland grasshoppers.

## 5. Conclusions

This study effectively evaluated the suitable habitat of an early dominant species in grassland regions of China by selecting optimized MaxEnt model parameters. *D. barbipes* is mainly distributed in the central and eastern parts of Inner Mongolia, with additional occurrences in the central Qilian Mountains of Gansu. The mean temperature of the coldest quarter and the coefficient of precipitation variation were identified as the most important driving factors influencing the distribution of *D. barbipes*, while altitude and human activities also played limiting roles. Under all future climate scenarios, the suitable habitat of *D. barbipes* is projected to shift northward, with stable areas mainly located in eastern Inner Mongolia. More pronounced changes are expected by the 2090s, with suitable habitats in the central and western regions nearly disappearing. Future research should incorporate a broader range of species traits and other biotic factors into model construction to better simulate realistic species distribution environments. This study essentially clarifies the response of the geographical distribution pattern of *D. barbipes* to climatic variables, providing a theoretical basis for pest prediction, forecasting, and management.

## Figures and Tables

**Table 1:** Environmental factors used for modeling potential suitable habitats.

Environmental Factor	Symbol
Mean diurnal temperature range (°C)	Bio2
Mean temperature of wettest quarter (°C)	Bio8
Mean temperature of coldest quarter (°C)	Bio11
Precipitation of wettest month (mm)	Bio13
Precipitation seasonality (%) (Coefficient of Variation)	Bio15
Precipitation of coldest quarter (mm)	Bio19
Elevation (m)	DEM
Aspect	Aspect
Slope (°)	Slope
Landuse type	Landuse
Soil pH	pH
Human footprint	HFP

**Table 2:** Percent contribution and cumulative contribution of environmental variables.

Variable	Cumulative Percentage (%)	Percentage of Variance (%)
bio11	83.50	83.50
bio15	93.00	9.50
DEM	96.90	3.90
HFP	97.90	1.00
Bio2	98.80	0.90
bio13	99.30	0.50

**Table 3:** Changes in the suitable habitat area of *D. barbipes* under future scenarios.

Climate Scenarios	Decades	Low Suitability (×10^4^ km^2^)	Middle Suitability (×10^4^ km^2^)	High Suitability (×10^4^ km^2^)
SSP126	2050s	9.65	11.13	4.27
	2070s	10.44	12.09	5.93
	2090s	9.05	9.90	7.26
SSP245	2050s	5.90	10.67	3.08
	2070s	5.19	11.84	2.50
	2090s	5.22	10.27	4.01
SSP585	2050s	8.23	10.19	6.26
	2070s	5.45	10.29	1.56
	2090s	5.81	8.11	1.55

## Data Availability

The original contributions presented in this study are included in the article/Supplementary Materials. Further inquiries can be directed to the corresponding author.

## Supplementary Materials

The following supporting information can be downloaded at: https://www.mdpi.com/article/10.3390/insects17060616/s1, Table S1: Locations of *Dasyhippus barbipes* occurrence points. Table S2: Environmental variables used for MaxEnt modeling. Table S3: Parameter optimization results. Table S4: Performance metrics (AUC and TSS) of 10 replicate MaxEnt runs. Figure S1: Results of the Jackknife test for environmental variable importance, including percent contribution and permutation importance.
